# Probiotic Influences on Motor Skills: A Review

**DOI:** 10.2174/1570159X21666230807150523

**Published:** 2023-09-25

**Authors:** Robert Lalonde, Catherine Strazielle

**Affiliations:** 1 Laboratory of Stress, Immunity, Pathogens (EA7300), Medical School, University of Lorraine, 54500, Vandœuvre-les-Nancy, France;; 2 CHRU Nancy, Vandœuvre-les-Nancy, France

**Keywords:** Lactobacilli, Bifidobacteria, Parkinson’s disease, multiple sclerosis, hepatic encephalopathy, motor coordination

## Abstract

The effects of probiotics have mostly been shown to be favorable on measures of anxiety and stress. More recent experiments indicate single- and multi-strain probiotics in treating motor-related diseases. Initial studies in patients with Parkinson’s disease and Prader-Willi syndrome are concordant with this hypothesis. In addition, probiotics improved motor coordination in normal animals and models of Parkinson’s disease, multiple sclerosis, and spinal cord injury as well as grip strength in hepatic encephalopathy. Further studies should delineate the most optimal bacterial profile under each condition.

## 1 INTRODUCTION

Probiotics are defined as bacteria with favorable physiological properties and are designated as psychobiotics when they produce behavioral outcomes [1-3], the most important of which include *Lactobacillus* (*L.*) *Bifidobacterium* (*B.*), *Streptococcus* (*S.*), *Escherichia* (*E.*), *Enterococcus* (*E.*), and *Clostridium butyricum* [4]. Most researchers in this field have focused attention on anxiety, stress, and depression *via* neurologic, endocrine, humoral, and immune systems [5-8]. The brain is affected in the absence of microbiota in germ-free animals. Effects on the brain were likewise noted after antibiotic administration. When bacterial strains, especially those deemed to be beneficial, were given to animals, neurobiological effects occurred, some of which also occurred in normal human subjects. Such studies were then extended to treat specific diseases.

The effects of gut microbiota were first evaluated in gastrointestinal diseases, cancer, obesity, and infections and then for neuropsychiatric symptoms [1-8]. In particular, the treatment of liver disease and its neurological complications with antibiotics led to an understanding of the role of microbiota in other neurological conditions. Since the gut is innervated by the vagus nerve, several experimenters have aimed at deciphering whether microbiotic actions are transmitted *via* this pathway. At the present time, effects on animal and human behavior have mostly been analyzed for cognition and emotion, to a lesser extent, sensorimotor functions. In the present review, we regard probiotic actions on sensorimotor functions under various neurologic conditions. Although most results are preliminary, this synthesis should be of use to guide future research.

## 2 NORMAL SUBJECTS

### 2.1 Normal or Preterm Human Subjects

Probiotics undergo changes across the lifespan [9], so there is interest in investigating their effects in children up to old age. In a preliminary investigation of this kind, *Bifidobacterium*, *Lactobacillus*, and *Coprococcus* strains were more abundant in 18-month-old infants above the median in the fine motor skill subscore of the Bayley-III battery of neuro-development [10]. It remains to be determined whether the administration of such strains increases motor skills in older children and adults.

In a double-blind, placebo-controlled, randomized trial, *B. infantis* BB-02 96579 + *B. lactis* BB-12 15954 + *S. thermophilus* TH-4 15957 had no effect on the Gross Motor Function Classification System score, the Bayley-III Motor Composite Scale, or the Movement Assessment Battery for Children in preterm children at 2-5 years of age [11]. In a review of five papers, including the preceding one, Upadhyay [12] concluded that probiotics had no effect on motor behaviors in preterm children.

### 2.2 Normal Animals

*L. plantarum* + *L. longum* increased latencies before falling from the rotorod in 1-month-old mice, either previously exposed to treadmill exercise or not [13]. The probiotic mixture increased cerebellar concentrations of gamma-aminobutyric acid (GABA), the cerebellum being particularly relevant in performing this type of task [14]. Likewise, *L. fermentum* JDFM216 increased latencies before falling from the rotorod and decreased foot slips from a stationary beam in 12-month-old mice [15]. These results lend credence to the hypothesis that probiotics facilitate motor coordination and thus may be of use in sporting events [16]. *L. plantarum* SPA3 + *L. rhamnosus* B-8238 counteracted the decrease in latencies before falling from the rotorod in antibiotic-induced dysbiosis in mice [17], indicating that individuals with suboptimal gut bacteria are more likely to improve with probiotics.

## 3 PARKINSON’S DISEASE

### 3.1 Patients with Parkinson’s Disease

Most probiotic studies on motor control have been conducted in the context of Parkinson’s disease. Based on gastrointestinal anomalies and alterations in the gut microbiota of such patients [18-22], studies have appeared on probiotic-induced improvements in patients’ constipation and neural functions, possibly *via* anti-inflammatory and antioxidant actions [23-30]. For example, *L. salivarius* LS01 and *L. acidophilus* LA02 DSM 21717 reduced proinflammatory cytokines as well as increased anti-inflammatory cytokines [31].

In an open-label, single-arm, baseline-controlled trial of patients with Parkinson’s disease, *L. plantarum* PS128 improved the total score of the Unified Parkinson’s Disease Rating Scale (UPDRS) and its akinesia subscore as well as a single index, mobility, and activities of daily living in the 39-item Parkinson’s Disease Questionnaire (PDQ-39) [32]. Likewise, *L. acidophilus* + *L. fermentum* + *L. reuteri* + *B. bifidum* improved the total score of the UPDRS in a double-blind trial [33].

### 3.2 Animal Models of Parkinson’s Disease

*L. acidophilus* + *L. plantarum* + *L. paracasei* + *L. delbrueckii* subsp. *bulgaricus* + *L. brevis* + *B. longum* + *B. breve* + *B. infantis* + *S. thermophilus*, designated as SLAB51, was given before a unilateral 6-hydroxydopamine (6-OHDA) injection in the mouse striatum and improved the use of the contralateral forepaw in the cylinder test and postural asymmetry in the elevated body swing test [34]. The supplement also counteracted 6-OHDA-induced decreases in tyrosine hydroxylase and dopamine transporter immunoreactivity in the substantia nigra, indicating preservation of dopaminergic neurons. Likewise, *L. salivarius* subsp. *salicinius* AP-32 increased locomotor speed and stride length while decreasing stance time in gait analyses of rats unilaterally injected with 6-OHDA along the nigro-striatal tract [35]. The probiotic also increased the number of tyrosine hydroxylase-positive neurons in the substantia nigra and the striatum. In a similar manner, *L. plantarum* PS128 improved step adjustment in gait analyses of rats unilaterally injected with 6-OHDA along the nigro-striatal tract and increased tyrosine hydroxylase-positive neurons in the substantia nigra [36]. In addition, 6-OHDA unilaterally injected in the substantia nigra followed by intraperitoneally administered apomorphine augmented contralateral rotations compared with controls and *L. acidophilus* + *L. reuteri* + *L. fermentum* + *B. bifidum* prevented these and increased the number of substantia nigra neurons destroyed by the neurotoxin [37]. *L. fermentum* U-21 alone counteracted the paraquat-induced increase in latencies before descending from a vertical pole and the decrease in the number of tyrosine hydroxylase-positive neurons in the substantia nigra [38].

In addition to 6-OHDA, probiotics appear effective in counteracting anomalies caused by the peripheral injection of another dopamine neurotoxin, methyl-phenyl-tetrahydro-pyridine (MPTP). Indeed, *L. plantarum* CRL 2130 + *S. thermophilus* CRL 807 and CRL 808 decreased latencies before descending from the vertical pole and latencies before traversing the horizontal stationary beam and increased tyrosine hydrolase-positive cell counts in the substantia nigra of MPTP-injected mice [39]. The same effects were found with *Clostridium butyricum* [40]. Moreover, a mixture of *L. lactis* MG1363 + glucagon-like peptide-1 (GLP-1) reduced latencies before descending from the vertical pole and increased open-field activity in mice injected with MPTP as well as counteracting declines in the number of tyrosine hydroxylase-positive neurons in the substantia nigra [41, 42].

In yet another Parkinsonian model, *L. rhamnosis* GG + *L.* rhamnosus + *L. plantarum* LP28 + *L. lactis* subsp. *lactis* + *B. bifidum* + *B. longum* reduced latencies before traversing the stationary beam and increased latencies before falling from the rotorod as well as improving gait patterns in MitoPark PD mice, characterized by inactivation of the *Tfam* (transcription factor A) mitochondrial gene in dopamine neurons [43]. The mixture also added protective actions on substantia nigra dopamine neurons as determined by the number of tyrosine hydroxylase-positive cells.

## 4 MULTIPLE SCLEROSIS

### 4.1 Patients with Multiple Sclerosis

Motor dysfunctions, sometimes leading to paresis, figure as important features in the symptomatology of multiple sclerosis [44]. Although there is doubt as to whether gut dysbiosis marks the cause or consequence of the disease, it has been considered a contributing factor [45-49]. Meta-analyses revealed the beneficial effects of probiotic supplementation on general health and depression in patients with multiple sclerosis [50-52]. In a double-blind trial, *L. reuteri* + *L. casei* + *L. plantarum* + *L. fermentum* + *B. infantis* + *B. lactis* improved the global score of the Expanded Disability Status Scale (EDSS) and the General Health Questionnaire-28 (GHQ-28) in patients with multiple sclerosis, the former featuring the ability to walk [53]. Further experiments must delineate whether this or other mixtures specifically improve motor scores.

### 4.2 Animal Models of Multiple Sclerosis

Antibiotics increased latencies before falling from the rotorod and improved axon damage in mice exposed to intracranial infection with Theiler’s murine encephalomyelitis virus (TMEV), a model of multiple sclerosis [54], lending credence to the hypothesis that gut microbiota influences demyelinating diseases. In further support of this hypothesis, *L. acidophilus* DSM 24735 + *L. paracasei* DSM 24734 +*L. plantarum* DSM 24730 + *L.* + *L. bulgaricus* DSM 24734 + *B. infantis* DSM 24737 + *B. longum* DSM 24736 + *B. breve* DSM 24732 + *S. thermophiles* DSM 24732, designated Vivomixx, increased latencies before falling from the rotorod and exhibited anti-inflammatory actions in TMEV-treated mice [55]. Both the Vivomixx mixture and a 3-strain mixture of *L. acidophilus* LA 201 + *L. salivarius* LA 304 *+ B. lactis* LA 304, designated Lactibiane iki, were examined in another murine model of multiple sclerosis: pertussis toxin-induced experimental autoimmune encephalomyelitis [56]. As in the previous study, both probiotic mixtures increased latencies before falling from the rotorod. On the contrary, neither *L. plantarum* nor *Bifidobacterium* B94 had any effect on swimming speed in rats exposed to ethidium bromide-induced demyelination [57]. In a third model of demyelination, the murine cuprizone-induced model, *L. casei* T2 augmented spontaneous alternation rates in a Y-maze [58], a measure of mental flexibility [59], so it remains to be determined whether these results can be generalized to motor coordination. More experiments are needed to determine whether motor signs should be included in the conclusions of a general review [60] and a meta-analysis [61] indicating probiotic-induced improvements on overall signs, body weight gain, and survival in experimental autoimmune encephalomyelitis.

## 5 SPINAL CORD INJURY

### 5.1 Patients with Spinal Cord Injury

Gut microbiota may be an important factor in mediating neural repair following spinal cord trauma [62]. In particular, spinal cord trauma may cause gut dysbiosis [63], so treating this condition becomes all the more relevant to the pathological process. Even with minimal actions on sensorimotor functions, psychobiotics may be of use in mitigating anxiety and depression [5-7].

### 5.2 Animal Models of Spinal Cord Injury

A mixture of broad-spectrum antibiotics worsened spinal cord pathology and slowed down recovery of locomotion in mice exposed to spinal cord trauma [64]. Like control mice, antibiotic-treated mice eventually exhibited consistent plantar stepping but without forelimb/hindlimb coordination and were more prone to paw anomalies and trunk instability. Conversely, *L. casei* + *L. plantarum* + *L. acidophilus* + *L. delbrueckii* subsp. *bulgaricus* + *B. longum* + *B. breve* + *B. infantis* + *S. salivarius* subsp. *thermophilus*, designated VSL#3, promoted spinal cord recovery relative to the vehicle-treated group by increasing the frequency of plantar stepping and forelimb/hindlimb coordination along with improving paw position and trunk stability. The product also reduced lesion volume as well as axon and myelin pathology at the injury site. These data should encourage further analyses in the patterns of dysbiosis seen after spinal cord injury so that optimal probiotics are proposed.

## 6 PRADER-WILLI SYNDROME

### 6.1 Patients with Prader-Willi Syndrome

Prader-Willi syndrome is a disorder caused by the deletion of paternally expressed genes at the Chr. 15q11.2-q13 locus, resulting in hypotonia, developmental delay, hyperphagia, obesity, and such neuropsychiatric signs as psychosis and compulsive behaviors [65]. Gut microbiotas have been linked to Prader-Willi syndrome [66], so a randomized, double-blind, placebo-controlled trial of *L. reuteri* LR-99 was conducted [67]. The probiotic improved the total score and fine motor skill subscore of the ASQ-3 test as well as the social communication subscore of the Gilliam Autism Rating Scale (GARS-3) while reducing body mass index.

### 6.2 Animal Models of Prader-Willi Syndrome

To our knowledge, a probiotic has yet to be examined in rodent models of Prader-Willi syndrome [68, 69].

## 7 HEPATIC ENCEPHALOPATHY

### 7.1 Patients with Hepatic Encephalopathy

Motor performance is impaired even in minimal hepatic encephalopathy [70]. Neurocognitive scores in patients with hepatic encephalopathy were correlated with the presence of bacterial DNA [71] in line with the hypothesis that favorable bacteria are of use in treating this condition. Indeed, probiotics are useful in treating biochemical anomalies of hepatic encephalopathy [72-75]. However, their use on human motor control has been left unexamined.

### 7.2 Animal Models of Hepatic Encephalopathy

The *E. coli* Nissle 1917 bacterium was genetically modified to consume and convert excessive ammonia to arginine and further modified to synthesize butyrate in rats with ligated bile ducts [76]. When the engineered product synthesized arginine + butyrate, forelimb and hind-limb grip strength improved, but not latencies before falling from the rotorod. The combination of the antibiotic rifaximin, along with the Vivomixx probiotic mentioned above, was given in rats with ligated bile ducts, a model of type C hepatic encephalopathy [77]. On magnetic resonance spectroscopy scans, duct ligation increased glutamine levels in the hippocampus and cerebellum, effects counteracted by rifaximin + Vivomixx, whereas the antibiotic alone was without effect. Duct ligation also decreased creatine levels in the cerebellum, an effect also counteracted by rifaximin + Vivomixx administration, while yet again, the antibiotic alone was without effect. Despite these favorable biochemical actions, there was no effect on bile duct ligation-induced hypoactivity in the open field. Further experimentation is required to determine whether probiotics specifically improve the sensorimotor impairment underlying hepatic encephalopathy.

## CONCLUSION

Positive results have been obtained on the UPDRS in patients with Parkinson’s disease given two *Lactobacillus* mixtures, data supported by those on three animal models measuring gait and motor coordination in vertical poles, stationary beams, and rotorod tests. One positive finding has been reported with *L. reuteri* on fine motor skills of the GARS-3 in Prader-Willi syndrome. But only experiments in mice or rats exist in regard to multiple sclerosis, spinal cord injury, and hepatic encephalopathy. In two models of multiple sclerosis, *Lactobacillus* combined with *Bifidobacterium* improved rotorod performance. Another mixture containing *Lactobacillus* and *Bifidobacterium* facilitated gait patterns in spinal cord injury. In hepatic encephalopathy, an improvement in grip strength was noted with *E. coli*. There is interest in extending such protocols with other motor-related diseases, such as spinocerebellar atrophy and amyotrophic lateral sclerosis.

## LIST OF ABBREVIATIONS

GLP-1: Glucagon-like Peptide-1
MPTP: Methyl-phenyl-tetrahydro-pyridine
PDQ-39: Parkinson’s Disease Questionnaire
TMEV: Theiler’s Murine Encephalomyelitis Virus
6-OHDA: 6-Hydroxydopamine
UPDRS: Unified Parkinson’s Disease Rating Scale
